# Unraveling the Glucosylation of Astringency Compounds of Horse Chestnut *via* Integrative Sensory Evaluation, Flavonoid Metabolism, Differential Transcriptome, and Phylogenetic Analysis

**DOI:** 10.3389/fpls.2021.830343

**Published:** 2022-02-03

**Authors:** Qinggang Yin, Yiding Wei, Xiaoyan Han, Jingwang Chen, Han Gao, Wei Sun

**Affiliations:** ^1^Artemisinin Research Center, Institute of Chinese Materia Medica, China Academy of Chinese Medical Sciences, Beijing, China; ^2^Beijing Botanical Garden, Institute of Botany, Chinese Academy of Sciences, Beijing, China; ^3^Institute of Food Science and Technology, Chinese Academy of Agricultural Sciences, Beijing, China

**Keywords:** *Aesculus chinensis*, kaempferol, astragalin, glycosides, glycosylation

## Abstract

The seeds of Chinese horse chestnut are used as a source of starch and escin, whereas the potential use of whole plant has been ignored. The astringency and bitterness of tea produced from the leaves and flowers were found to be significantly better than those of green tea, suggesting that the enriched flavonoids maybe sensory determinates. During 47 flavonoids identified in leaves and flowers, seven flavonol glycosides in the top 10 including astragalin and isoquercitrin were significantly higher content in flowers than in leaves. The crude proteins of flowers could catalyze flavonol glucosides' formation, in which three glycosyltransferases contributed to the flavonol glucosylation were screened out by multi-dimensional integration of transcriptome, evolutionary analyses, recombinant enzymatic analysis and molecular docking. The deep exploration for flavonol profile and glycosylation provides theoretical and experimental basis for utilization of flowers and leaves of *Aesculus chinensis* as additives and dietary supplements.

## Introduction

The Chinese horse chestnut (*Aesculus chinensis*) is a deciduous tree native to Qinling Mountains of China. The seeds of *A*. *chinensis* are used as a source of starch via alkali treatment or high-temperature detoxification, as well as a source of escin, which is a pentacyclic triterpenoid saponin (Zlatanov et al., 2013; Cheng et al., 2018; Zhang et al., 2020a). The seeds of the Japanese horse chestnut (*A. turbinata*) have been an important food resource since ancient times and are a good source of flavonol O-glycosides, which possess considerable antioxidative capacities for use as food additives and dietary supplements (Kimura et al., 2017). Moreover, some *Aesculus* spp. contain components that are beneficial to human health, especially flavonoids (Wei et al., 2004; Kapusta et al., 2007; Zlatanov et al., 2013; Oszmiański et al., 2014; Zhang et al., 2020b; Jarzebski et al., 2021). Various herbal tea products have been developed from the flowers of the European horse chestnut (*A. hippocastanum*) and the buds of *A. chinensis*. However, as compared with the widespread use of the seeds of *A. chinensis*, the flowers and leaves have been mostly ignored due to the lack of detailed analysis of key compounds that are potentially beneficial to human health.

Flavonoids, which are abundant in various foods of plant origin, possess antioxidative and anticancer activities, but also contribute to the taste of tea, ginkgo leaves, tartary buckwheat, ginseng leaves, and lotus leaves or seeds (Cui et al., 2016; Su et al., 2017; Cao et al., 2019; Yin et al., 2020; Feng et al., 2021). The sweet aftertaste of tea is due to the contents of (-)-epigallocatechin and (-)-epicatechin, while flavonol glycosides are associated with the astringency and bitterness of tea infusions (Scharbert et al., 2004; Scharbert and Hofmann, 2005; Cao et al., 2019; Dong et al., 2019; Guo et al., 2021). The seeds of *A. chinensis, A. hippocastanum, A. carea*, and *A. turbinate* contain various flavonol glycosides (Kapusta et al., 2007; Kimura et al., 2017; Cheng et al., 2018; Zhang et al., 2020a). Glycosides of quercetin and kaempferol have been identified in the leaves of *A. hippocastanum* and *A. carea* (Oszmiański et al., 2014). Kaempferol, which exerts defensive effects against oxidative damage to the brain tissues induced by various types of agents, can cross the blood brain barrier and reduce neuronal damage (Chen and Chen, 2013). Quercetin is most commonly used for conditions of the heart and blood vessels and to prevent cancer (Xu et al., 2018a). The leaves and flowers of *A. chinensis* would been explored as potential food sources if the flavonoid profiles were similar to that of same genus reported.

Flavonoid glycosides are catalyzed by uridine diphosphate glycosyltransferases (UGTs), which transfer glycosyl moieties from UDP sugars to a wide range of acceptor molecules (Li et al., 2021). Different glycosyltransferases catalyze the formation of various flavonoid mono-glycosides. FaGT1 (*Fragaria* x *ananassa*) derived from the strawberry (Griesser et al., 2008), GmUGT88A13 (*Glycine max*) from the soybean (Yin et al., 2017a), and LjUGT72AD1 from *Lotus japonicus* are reported to glucosylate the 3-OH group of flavonols (Yin et al., 2017b). In tartary buckwheat (*Fagopyrum tataricum*), FtUGT73BE5 is the key molecule in the first step glycosylation of rutin (Yin et al., 2020). In ginseng (*Panax gensing*), PgUGT92A10 and PgUGT94Q4 contribute to the formation of kaempferol 3-O-glucoside, as determined by differentiated data-independent acquisition proteomics and phylogenetic analysis (Yin et al., 2021). In ginkgo (*Ginkgo biloba*), GbUGT716A1 possesses unique activities toward flavanol gallates and conveys significant bioactivity beneficial to human health (Su et al., 2017). However, relatively few reports have been identified and characterized as UGT proteins involved in the biosynthesis of flavonoid glycosides in *Aesculus* genus including *A. chinensis*.

In this study, sensory evaluation indicated that herbal tea produced from *A. chinensis* had an excellent taste. Flavonoid profiles of the leaves and flowers revealed that quercetin or kaempferol glycosides may contribute to the strong astringency of herbal tea. Crude protein enzymatic testing indicated that UGTs may contribute to the abundant accumulation of astragalin (kaempferol 3-O-glucoside) in flowers. The sequenced transcriptomes of the flower and seed of *A*. *chinensis* were used to construct a database of AcUGTs. Screening of 170 AcUGTs identified three that participate in the formation of isoquercitrin (quercetin 3-O-glucoside) and astragalin, which are abundant in the flowers of *A. chinensis*. These results demonstrated that these AcUGT genes, which are responsible for the accumulation of flavonol glycosides in *A. chinensis*, are important to the flavor of herbal tea, and should prove helpful for the development of healthy foods derived from *A. chinensis*.

## Materials and Methods

### Plant Materials and Chemicals

*A. chinensis* leaves, flowers, and seeds were obtained from the Institute of Medicinal Plants of the Chinese Academy of Medical Sciences (Beijing, China), then immediately washed, frozen in liquid nitrogen, and stored at −80°C for later use. Some leaves and flowers were naturally dried to produce an herbal tea. Green tea made in spring of 2021 was purchased in the market.

The pMAL-c2x expression vector was preserved by the Biotechnology Center of the Institute of Chinese Materia Medica, China Academy of Chinese Medical Sciences (Beijing, China). UDP-glucose was purchased from Sigma-Aldrich Corporation (St. Louis, MO, USA). Standard flavonols (kaempferol, quercetin, quercitrin, isoquercitrin, and astragalin) were purchased from Shanghai Yuanye Biotechnology Co., Ltd. (Shanghai, China). Chemicals were either analytically pure or high-performance liquid chromatography grade.

### Sensory Evaluation of Green Tea and Herbal Tea Infusions

According to Cao et al. method (Cao et al., 2019), green tea or herbal tea (2g) was extracted with distilled boiled water (1:50, w/w), standing for 5 min. Then, infusions of green and herbal tea (about 40°C) were evaluated by a trained team of nine panelists (three men and six women, 23–38 years old). Each member of the team graded the bitterness, astringency, and sweet aftertaste of the tea using a modified nine-point scale, where 8–10 “extremely strong,” 6–8 “strong,” 4–6 “neutral,” 2–4 “weak,” and 0–2 “very weak.” For the sensory evaluation, a 50 mL aliquot of the sample solution was served in a cup and the intensity of bitterness was recorded by tasting the sample solution while swirling in the mouth for 7–8 s. Then, the solution was spat out and the intensity of astringency was recorded within 3–4 s. After recording the astringency, the examiners were instructed to rinse their mouths with pure water and record the intensity of the sweet aftertaste within 4–5 s. There was a 5 min interval between tests. The results were analyzed statistically to determine significant differences between the mean scores of the different samples. Each evaluation was repeated three times with the order of the samples randomized in each test.

### Analysis of Flavonoid Profiles and Flavonol Metabolites by Ultra-High-Performance Liquid Chromatography–Tandem Mass Spectrometry (UPLC/MS/MS)

The freeze-dried samples were crushed with a mixer mill for 240 s at 45 Hz. Then, 10 mg of each powdered sample were transferred into a 5 mL Eppendorf tube and extracted with 3 mL of 75% methanol/1% acetic acid. After vortexing for 30 s, the samples were homogenized at 40 Hz for 4 min and sonicated for 10 min in an ice-water bath. The homogenization and sonication steps were repeated three times. Following centrifugation at 13, 800 × *g* for 15 min at 4°C, a 2.5 mL aliquot of the supernatant was dried under a gentle flow of nitrogen gas. The residue of each sample was reconstituted in 1.0 mL of 50% methanol/0.1% formic acid, then vortexed for 30 s, ultra-sonicated for 15 min in an ice bath, and centrifuged at 13, 800 × *g* for 15 min at 4°C. The resulting supernatants were filtered through a membrane with a pore size of 0.22 μm, transferred into 2 mL glass vials, and stored at −80°C until analysis by UPLC-MS/MS. A quality control sample was prepared by mixing equal volumes of the supernatants of all samples.

Herbal teas made from flowers and leaves were analyzed using an Acquity UPLC system (Waters Corporation, Milford, MA, USA) equipped with a BEH C18 column (1.7 μm, 2.1 × 150 mm). The samples were separated with a gradient composed of acetonitrile (A) and 0.1% formic acid water (B) under the following parameters: injection volume, 2 μL; column temperature, 40°C, and flow rate, 0.3 mL·min^−1^. The first equilibrium at 90% B was maintained for 0.5 min. Then, the analytes were eluted using a linear gradient of 90% → 40% B for 0.5–15 min and washed for 3 min with 2% B. The final equilibrium at 90% B was maintained for 2 min. The total running time was about 20 min with the use of a QTRAP^®^ 6500+ LC-MS/MS System (AB SCIEX, Concord, ON, Canada). Typical ion source parameters were: positive (+) and negative (–) mode; ion spray voltage, +5000/−4500 V; curtain gas, 35 psi, temperature, 500°C; ion source gas 1, 55 psi; and ion source gas 2, 60 psi.

The *A. chinensis* leaves, flowers, and seeds stored in −80°C were extracted as described above to quantify the target flavonols. Astragalin, isoquercitroside, quercitrin, quercetin, and kaempferol were detected using an Agilent 1290 Infinity UPLC system coupled an Agilent 6470 Triple Quadruple LC/MS system (Agilent Technologies, Inc., Santa Clara, CA, USA) equipped with an Agilent ZORBAX RRHD Eclipse Plus C18 column. The samples were separated with a gradient composed of acetonitrile (A) and 0.1% formic acid water (B), using eluted program, 0–7 min, 95% → 5% B; 2 min for wash with 100% A, and 1 min for equilibrium with 95% B. Under the following parameters: injection volume, 2 μL; wavelength, 254 nm; column temperature, 35°C; and flow rate, 0.3 mL·min^−1^. The mass spectrometer was operated in negative ion mode with the following parameters: sheath gas temperature, 300°C; gas flow, 5.0 L min^−1^; nebulizer gas, 45 psi; capillary voltage, 3500 V; nozzle voltage, 500 V, and delta electron multiplier voltage, 200 V. The ion conditions for detection by UPLC-MS were the same as described above and metabolites were detected in multiple reactions monitoring mode. Data analyses were conducted with MassHunter qualitative analysis software (version B.07.00; Agilent Technologies).

### Crude Protein Extraction and Enzymatic Analysis of *A. chinensis* Flowers

Freshly harvested flowers were ground twice using a freeze grinder (JX-2016, 50 Hz) for 30 s and total protein was extracted (0.1 g ml^−1^) in ice-cold extraction buffer (100 mM Tris at pH 7.0, 10 mM dithiothreitol, 0.1 mM phenylmethylsulphonylfluoride, 0.5% polyvinyl pyrrolidone). The extract was centrifuged at 13, 800 × *g* for 10 min at 4°C, and the supernatant was concentrated with an ultrafiltration device (30 KDa) and substitution was conducted with an enzymatic reaction buffer at 4°C. The crude extract was then assayed for enzyme activity in 100 μL reaction volumes consisting of 10 mM dithiothreitol, 50 mM Tris-HCl (pH 7.0), 2 mM UDP-glucose, and 100 μM sugar acceptor. After 30 min of incubation at 37°C, the reactions were terminated with ethyl acetate (400 μL), then, the organic phase was collected after centrifuging at 13, 800 × g for 5 min, passed through a 0.22 μm filter membrane, evaporated to dryness, and re-dissolved with methanol (100 μL). A 2 μL aliquot was analyzed using a UPLC-MS/MS system as described above.

### Differential Transcriptome and Phylogenetic Analysis

The expression patterns of AcUGT genes associated with the flavonol glycoside biosynthetic pathway were examined, ~33.3 Gb of clean data were downloaded from the National Center for Biotechnology Information (NCBI) database (SRR8073719–SRR8073722). RNA-Seq reads from each tissue were assembled using Trinity software with no reference genome. The expression level of each gene was evaluated with the Fragments Per Kilobase per Million mapped fragments method and calculated using Tophat and Cufflinks (version 2.1.1) with default parameters.

Multiple sequence alignments of the deduced amino acid sequences were conducted using DNASTAR bioinformatics software (https://www.dnastar.com/). The predicted amino acid sequences of the UGTs were aligned using the Clustal X2 multiple sequence alignment algorithm (http://www.clustal.org/) before phylogenetic analysis. A neighbor-joining phylogenetic tree was constructed with 1000 boot-strap replicates using Molecular Evolutionary Genetics Analysis 4.0 software (https://www.megasoftware.net/).

### Enzymatic Assay of AcUGT Recombinant Proteins and Product Identification

Primers for the 30 AcUGT genes were designed based on the AcGTs database of transcriptomes assembled without a reference genome. Mixed cDNAs from the leaves, flowers, and seeds of *A. chinensis* were used for gene amplification. The PCR products were purified and digested using the corresponding restriction enzymes, then ligated to the pMAL-c2x vector (New England BioLabs, Ipswich, MA, USA) and digested with the same restriction enzymes for expression of recombinant proteins in *Escherichia coli*.

Recombinant UGT proteins in *E. coli* were obtained as Yin et al. (2021). The crude recombinant proteins were used for enzymatic activity testing. The reaction system and conditions were same as for the crude proteins of *A. chinensis*, while the concentration of the tested acceptor substrates (0–400 μM) and purified enzymes (5 μg) was used for kinetic analysis. Further UPLC- MS/MS analysis was conducted as above mentioned. The kinetic parameters *K*m and *K*cat were calculated with the Hyper 32 program (http://hyper32.software.informer.com/).

### Homology Modeling and Docking Statistic

Homology models of the AcUGT1, AcUGT22 and AcUGT26 were constructed using the three-dimensional structure of UGT74AC2 (PDB ID: 7BV3; https://www.rcsb.org/, *Siraitia grosvenorii*), UGT85H2 (2PQ6, *Medicago truncatula*) and PaGT3 (6LZX, *Phytolacca Americana*) as a template respectively, by the SWISS-MODEL server (http://swissmodel.expasy.org) (Li et al., 2007, 2021; Maharjan et al., 2020). UDP-glucose or UDP-rhamnose and quercetin were docked with the model structure of AcUGTs using the Igemdock 2.1 docking program (http://gemdock.life.nctu.edu.tw/dock/igemdock.php). The docking result with UDP-glucose or UDP-rhamnose and quercetin was visualized with the Pymol molecular graphics system (http://www.pymol.org).

### Statistical Analysis

Statistical analyses were performed using Excel software (Microsoft Corporation, Redmond, WA, USA). The unpaired, two-legged Student's *t-*test was used to identify significant differences. A probability (*p*) value of < 0.05 was considered statistically significant. Data are presented as the mean ± standard deviation (n ≥ 3).

## Results

### The Taste of *A. chinensis* Herbal Tea and Metabolic Profiles of Flavonoids

**Figure 1:**
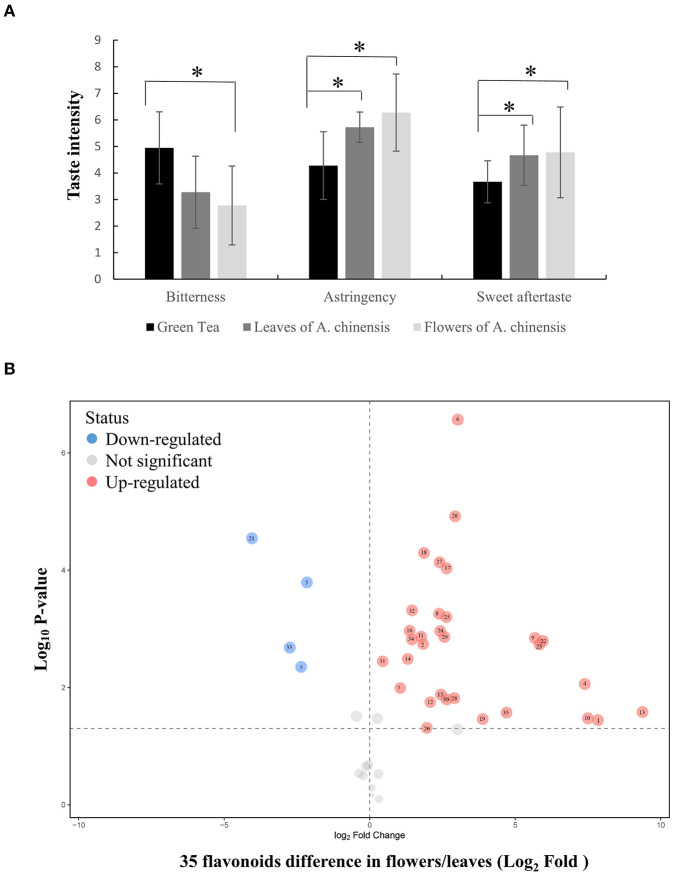
Sensory evaluation and flavonoid profile of *A. chinensis* herbal tea. **(A)** The taste intensity of green tea, herbal tea madding from flowers and leaves of *A. chinensis*. **(B)** The flavonoid differential analysis between flowers and leaves. 1, (-)-Epigallocatechin; 2, Apigenin; 3, Astilbin; 4, Astragalin; 5, Avicularin; 6, Chalconaringenin; 7, Chrysin; 8, Cianidanol; 9, Cynaroside; 10, Dihydromyricetin; 11, Engeletin; 12, Galangin; 13, Gallocatechin; 14, Genkwanin; 15, Hyperoside; 16, isoliquiritigenin; 17, Isoquercitrin; 18, Isorhamnetin; 19, Isorhamnetin-3-O-nehesperidine; 20, Isosakuranetin; 21, Kaempferitrin; 22, Kaempferol; 23, Kaempferol-3-O-rutinoside; 24, Myricitrin; 25, Narcissoside; 26, Naringenin; 27, Narirutin; 28, Phloretin; 29, Pinocembrin; 30, Procyanidin B1; 31, Procyanidin B2; 32, Quercetin; 33, Quercitrin; 34, Rutin; 35, Tiliroside. Data were statistically evaluated using Student's *t* test (*P < 0.05).

To explore the possibility of utilizing the whole *A. chinensis* plant, the leaves and flowers were naturally dried to produce herbal tea. Sensory evaluation of green tea and *A. chinensis* herbal tea infusions indicated that *A. chinensis* flowers produced an infusion that was obviously less bitter with a superior sweet aftertaste and stronger astringency (>6 points) than the green tea. Notably, the bitterness of the flower infusion was about half of that of green tea (Figure 1A). The astringency of the leaf infusion was also significantly stronger than the green tea, although all scores were between 4 and 6 (neutral level). The astringency results suggested that flavonoids may be abundant in the leaves and flowers of *A. chinensis*, which present potential materials for the production of foods to maintain health.

The flavonoid profiles of herbal tea made from *A*. *chinensis* leaves and flowers were investigated to identify flavonoids that determine the degree of astringency. As shown in Figure 1, Supplementary Figure 1, there were significant changes in the expression patterns of 35 of 47 flavonoids in *A*. *chinensis* flowers vs. leaves (31 flavonoids in flowers were higher than those in leaves, only four flavonoids were lower). In flowers, seven of the top 10 peak areas were flavonols other than (-)-epigallocatechin, procyanidin B2, and cynaroside (luteolin-7-glucoside). The peak area of astragalin was the highest at over 160-fold as compared to that in leaves. Similarly, eight of the top 10 peak areas of leaves were flavonols other than procyanidin B2 and cynaroside, with quercitrin (quercetin 3-O-rhamnoside) having the highest value. Gallocatechin (-)-epigallocatechin were also accumulated in flowers, especially the relative amount of (-)-epigallocatechin was over 229-fold greater than that of leaves, however, we did not detected epigallocatechin-3-gallate and epicatechin-3-gallate in flowers or leaves, which are the key compounds contributed to overall astringent taste of tea infusions (Scharbert and Hofmann, 2005). Collectively, these results show that flavonol glycosides are abundant in *A. chinensis* leaves and flowers, especially flavonol mono-glucosides, which are key to the astringent taste compounds of tea (Scharbert et al., 2004).

**Figure 2:**
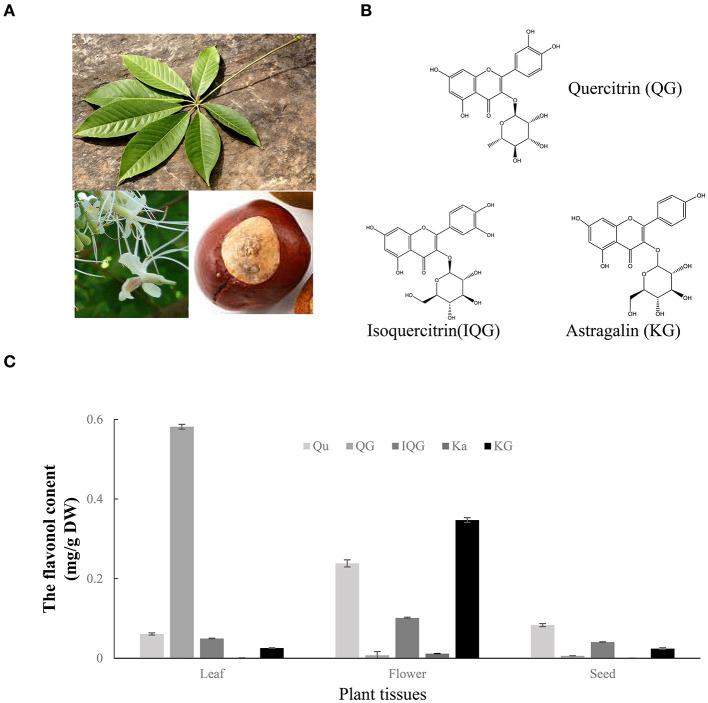
Flavonol glycosides in different tissues of *A. chinensis*. **(A)** Images of the seeds, flowers, and leaves of *A. chinensis*. **(B)** The molecular structures of three main flavonol glycosides. **(C)** The contents of flavonols in the three tissues. Qu, quercetin; Ka, kaempferol; IQG, isoquercitrin; KG, astragalin; QG, quercitrin.

To investigate the formation process of flavonol mono-glucosides *in planta*, the flavonols (kaempferol and quercetin) and three flavonol glucosides (i.e., astragalin, isoquercitrin, and quercitrin) were quantified in fresh seeds, flowers, and leaves of *A*. *chinensis* (Figures 2A,B). As shown in Figure 2C, Similar to results from herbal tea, tested flavonol or flavonol glycosides in the flowers higher than that in leaves, unless quercitrin which mainly accumulate in leaves reaching 0.59 mg/g. The astragalin content reached to 0.352 mg/g in flowers, which was 14- and 16-fold greater than that of the leaves and seeds, 0.025 and 0.022 mg/g respectively. The isoquercitrin content of the flowers was 0.103 mg/g, which was 2.1- and 2.7-fold greater than that of the leaves and seeds, respectively. The quercetin content of the flowers was 0.229 mg/g, which was 3.8- and 3.1-fold greater than that of the leaves and seeds, respectively. Interestingly, the content of quercetin in flowers was higher than isoquercitrin and quercitrin, and more than half of the content of astragalin. Flavonol glucosides which may play important roles in the enhanced taste, lower bitterness, and higher astringency of *A*. *chinensis* herbal tea were highly accumulated in the flowers, suggesting that some enzymes contributed to the formation of flavonol glucosides.

### Crude Proteins in Fresh Flowers Catalyzed the Formation of Flavonol Glucosides

**Figure 3:**
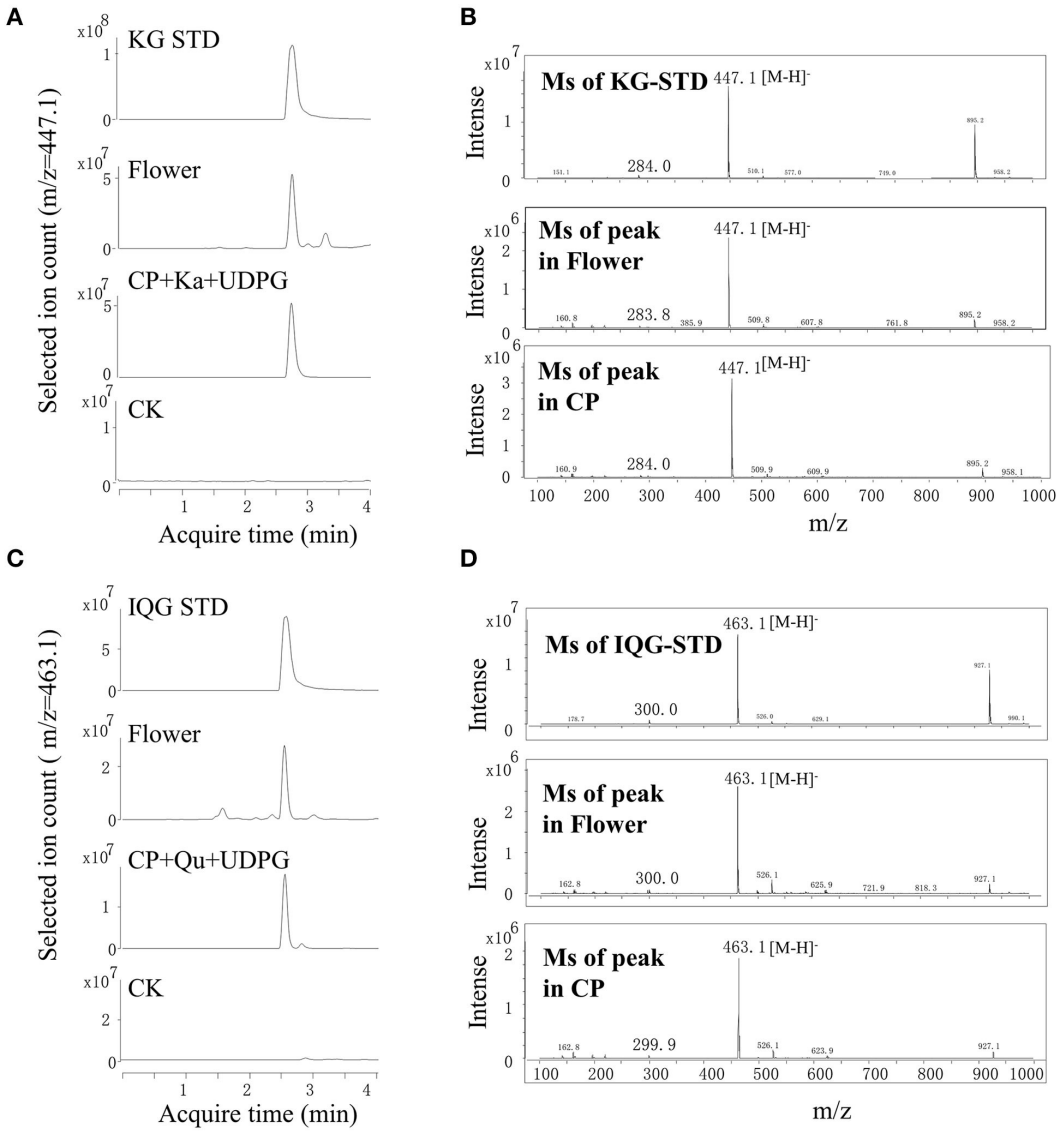
Crude proteins from flowers catalyzed the formation of astragalin and isoquercitrin *in vitro*. **(A,B)** UPLC-MS chromatograms of extracts from flowers and crude proteins (CP) with kaempferol (Ka). **(C,D)** UPLC-MS spectra of extracts from flowers and CP with isoquercitrin (IQG).

Enzymes contained in the flowers involved in the glycosylation of flavonol in *A. chinensis* were investigated. Although the leaves contained quercetin 3-O-rhamnose, the lack of UDP-rhamnose (a sugar donor) limited the further investigation. Crude proteins extracted from fresh flowers were used, UDP-glucose acted as a sugar donor and quercetin or kaempferol as a sugar acceptor (Figure 3). As compared with the standard of kaempferol 3-O-glucoside, the enzymatic product obtained with UDPG and kaempferol contained a common fragment ([M-H]^−^, 447.1). The MS spectrum and acquisition time of the new product were identical to those of the *A. chinensis* flower (Figures 3A,B). With quercetin as the sugar acceptor and UDPG as the sugar donor, the result was similar, as the crude proteins isolated from the flowers could catalyze the formation of quercetin 3-O-glucoside ([M-H]^−^, 463.1, Figures 3C,D). These enzymatic activities were compatible with the accumulation of flavonol mono-glucosides in flowers. The crude proteins of the *A. chinensis* flower involved in flavonol glycosylation suggested that some UGT genes should highly expressed in these tissues.

### Differential Transcriptome, Phylogenetic Analysis, and Enzymatic Testing to Screen UGTs

Since the genome of *A. chinensis* has not yet been sequenced, six transcriptome sequences were retrieved from the NCBI database to assess candidate UGT genes. Based on the transcriptome data, 170 genes annotated as GTs were used to construct an AcGT database for expression profile analyses (Supplementary Figure 2; Supplementary Data 1). Hierarchical clustering analysis showed that 105 and 65 GT genes were highly expressed in the flowers and seeds of *A. chinensis*, respectively. Some GTs were predicted to have other functions, such as the formation of pectin in the cell wall. Unigene37810, Unigene38108, Unigene15907, and Unigene16155 were predicted to code for galacturonosyltransferases, which contribute to pectin formation in the cell wall. Unigene13904, which was only expressed in the flowers, was annotated as O-fucosyltransferase 28. Unigene29217 and Unigene17314 were predicted to code for cellulose synthase and N-acetylglucosaminyltransferase, respectively. These GTs greatly interfered with the selection of candidate genes.

**Figure 4:**
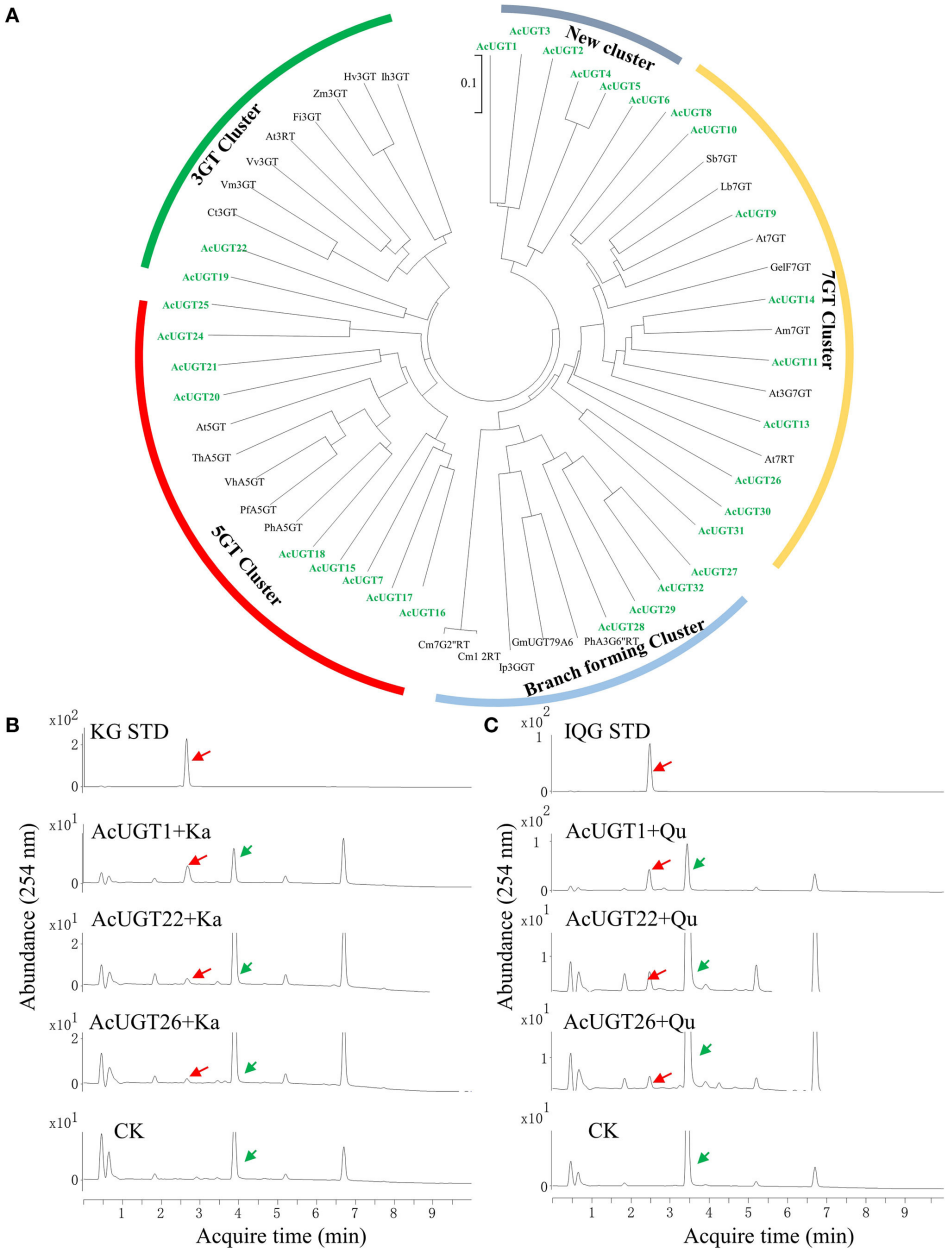
A phylogenetic tree and enzymatic analysis to screen for flavonol glucosyltransferases. **(A)** A phylogenetic tree of 30 AcUGTs and reported UGTs, sequence of reported UGTs were from Yin et al. (2021). **(B)** The UPLC spectra of AcUGT recombinant proteins with UDPG and kaempferol (Ka). **(C)** The UPLC spectra of AcUGT recombinant proteins with UDPG and quercetin (Qu). The green arrows indicate substrates, while the red arrows indicate products. KG STD, astragalin standard; IQG STD, isoquercitrin standard. CK means control, the enzymatic activity of the boiled protein encoded by empty-vector, substrates and UDP-glucose.

To precisely screen the AcUGTs involved in the formation of flavonoids, the PSPG box was used to manually annotate the AcGT database. Finally, 30 candidate AcUGTs were obtained for further study. Twenty-nine candidate AcUGTs consisted of 44 amino acids with glutamine as the last amino acid of the PSPG box, only the last amino acid of AcUGT29' PSPG box is aspartic acid (Supplementary Figure 3A). Glutamine is the key determinant of whether UGTs use UDP-glucose as a sugar donor (Yang et al., 2018). Phylogenetic analysis showed that 55 UGTs, including candidate AcUGTs and 25 previously identified UGTs, clustered into five major branches, which may provide important clues about the enzymatic functions of candidate UGTs (Figure 4A). The first cluster, represented by ZmF3GT, consisted of 10 UGTs, including AcUGT19 and AcUGT22, that catalyze the glycosylation of the 3-OH sites of flavonoids. The second cluster contained five UGTs that glycosylate the 5-OH sites of flavonoids, such as PfA5GT, and including nine AcUGTs. This finding suggests that AcUGTs in cluster two may catalyze the glycosylation of the 5-OH sites of flavonoids. In the third branch, seven AcUGTs were clustered with seven UGTs that glycosylate the 7-OH sites of flavonoids, while AcUGT26, AcUGT30, and AcUGT31 are located in the side branch in the cluster. Another branch formed a cluster of nine UGTs that catalyze multistep glycosylation of flavonoids, such as Ip3GGT. AcUGT1-6 formed a new branch, suggesting that this protein may be involved in different functions. To explore the glycosylation of the 3-OH sites of flavonols, the members of the 3GT cluster and some AcUGTs wth unique positions in phylogenetic tree, such as AcUGT26 or AcUGT1-6, were selected for further enzymatic experimentation.

To investigate the glycosylation of flavonoids, especially kaempferol and quercetin, the 30 AcUGTs were tried to clone, finally three AcUGTs were cloned and further identified using a prokaryotic system (Supplementary Figure 3B; Supplementary Data 1). UDP-glucose was used as a glycosyl donor for the enzymatic activity test with kaempferol or quercetin as the substrate. AcUGT1, AcUGT22, and AcUGT26 could catalyze the hydroxyl glucosylation of quercetin or kaempferol with the use of UDP-glucose as the glycosyl donor (Figures 4B,C). Analysis of the enzymatic products by UPLC-MS showed that recombinant AcUGT1, AcUGT22, and AcUGT26 produced new products with kaempferol and quercetin (Supplementary Figures 3C,D; Supplementary Figure 4). The m/z value of the glycosylation products was increased by 162 (the [M-H]^−^ m/z value of kaempferol and quercetin is 447.1 and 463.1 respectively). Based on the standard products of quercetin 3-*O*-glucoside and kaemperol 3-*O*-glucoside, the glycosylation product of both AcUGTs was flavonol 3-*O*-glucoside.

### Characterization of AcUGTs

**Figure 5:**
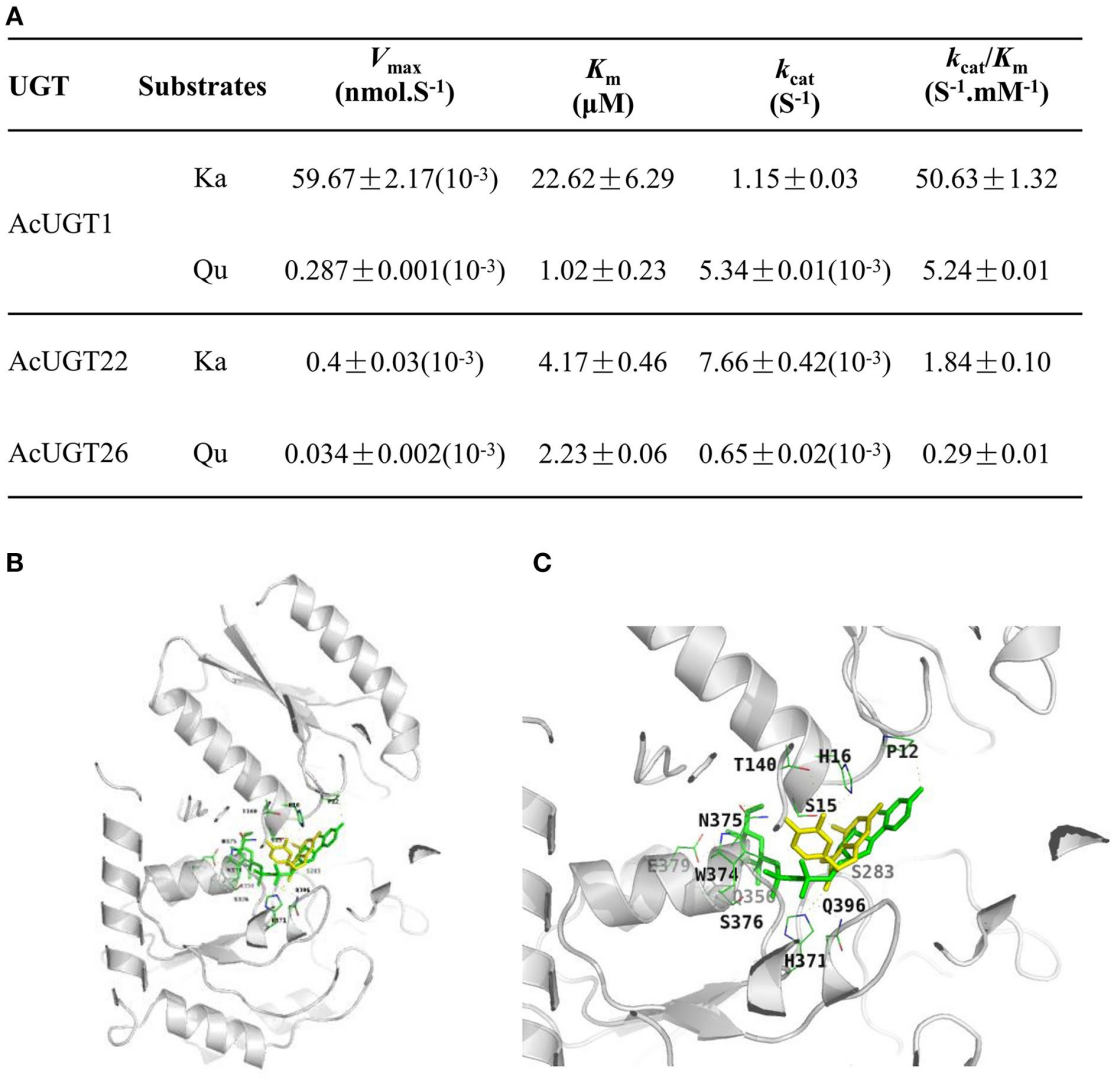
Characterization of AcUGTs. **(A)** Kinetic characteristics of three AcUGTs. **(B)** A ribbon diagram of UDPG and quercetin with AcUGT1. **(C)** An enlarged ribbon diagram of the active docking domain. UDPG is indicated in green and quercetin in yellow.

Enzyme kinetics analysis revealed that the catalytic efficiency of AcUGT1 (*k*_cat_/*K*_m_ value of kaempferol and quercetin was 50.63 and 5.24 S^−1^·mM^−1^, respectively) was much higher than that of AcUGT22 with kaempferol or AcUGT26 with quercetin. Moreover, AcUGT1 was more efficient with kaempferol than quercetin over 9.6-fold (Figure 5A), contrastly, the affinity of AcUGT1 to kaempferol was just about 4.5% of that to quercetin. AcUGT22 possessed a higher affinity toward kaempferol than AcUGT1 (*K*_m_ value of AcUGT22 and AcUGT1, 4.17 vs. 22.62 μM, respectively), while the affinity of AcUGT26 toward quercetin is lower than AcUGT1 (*K*_m_ value of AcUGT26 and AcUGT1, 1.02 vs. 2.23 μM, respectively). Although the three AcUGTs could act as a common substrate of the 3-OH sites of flavonols, differences in the affinity or efficiency toward different flavonols were mainly due to structural differences.

As shown in Figures 5B,C, the substrate quercetin (Qu) and the glycosyl donor UDP-glucose (UDPG) are both in the pocket created for binding with substrates in the model of AcUGT1. Several residues of AcUGT1, including Pro12, Ser15, His16, Thr140, Ser283, His371, and Asn375, form hydrogen bonds with quercetin, while Pro12, Ser15, Ser283, Gln356, His371, Gly373, Trp374, Asn375, Ser376, Glu379, and Gln396 form hydrogen bonds with UDPG. These residues could be important for the catalysis activity of AcUGT1. Based on the total energy for docking of kaempferol by the three AcUGTs (Supplementary Table 3), AcUGT1 required the lowest amount of energy and, thus, might be the most efficient, which is in agreement with the enzymatic activity results (*k*_cat_/*K*_m_ value of AcUGT1 for kaempferol was the highest at 50.63 S^−1^·mM^−1^).

To identify the putative glycosyltransferases involved in the formation of quercetin 3-O-rhamnose, the docking result of AcUGT26 showed that quercetin and UDP-rhamnose (UDPR) were located in the cleft of the AcUGT26 model, suggesting that AcUGT26 may contribute to the rhamnosylation of quercetin (Supplementary Figure 5C). As compared with AcUGT26 docking with UDPG or quercetin, fewer residues of the PSPG box of AcUGT26 formed hydrogen bonds with UDPR or quercetin, only Asn361, Glu381, and Gln382. However, these active sites in docking AcUGT26-UDPR-quercetin are similar to AcUGT1-UDPG-quercetin (Asn375, Glu379, and Gln396) and AcUGT26-UDPG-quercetin (Asn361, Glu381, and Gln382), indicated that AcUGT1 and AcUGT26 possess the same model for interacting substrates and sugar donor in spatial structure.

## Discussion

### Metabolic Profiles of *A. chinensis* Flavonoids Revealed Special Spatial Distributions

There are various metabolic patterns of flavonoids in plants. For example, the leaves of *Ginkgo biloba* are rich in flavonol polyglycosides and acylated flavonoids (Su et al., 2017). In tea leaves, flavonols mainly exist in the form of O-glycosides with a glycoside moiety at the C-3 position of aglycones (e.g., quercetin, kaempferol and myricetin), which account for ~13% of total tea polyphenols in fresh tea leaves following the most abundant flavonoid subclass flavanols (primarily catechins) (Xu et al., 2018b; Zhuang et al., 2020). Soybean seeds contain an abundance of isoflavones, while flavonol di- or multi-glycosides are the main form in leaves (Yin et al., 2017a). The leaves of *Andrographis paniculata* contain andrographolides, while the roots are enriched with flavonoids (Sun et al., 2019). Ginseng leaves contain high concentrations of flavonoids, including kaempferol 3-O-glucoside and panasenoside, which are kaempferol di-glycosides, while the roots contain trace amounts of flavonoids (Yin et al., 2021). The seeds of *A. chinensis* contain flavonol monoglycosides, polyglycosides, and acyl flavonoids (Zhu et al., 2006). Quercitrin was reported to mainly accumulate in leaves of *A. hippocastanum* and *A. carea* (Oszmiański et al., 2014), similarly, the flavonoid profile of *A. chinensis* also reveals quercitrin abundant in its leaves. Furthermore, the flowers of *A. chinensis* was firstly reported to contain high concentrations of flavonol mono-glucosides which contribute to the low bitterness, strong astringency of infusion (Scharbert and Hofmann, 2005).

### Flavonols Exploration Herbal Tea Promotes the Whole Plant Utilization of *A. chinensis*

The seeds of *A. chinensis* after alkali treatment or high-temperature detoxification treatment were intake as strarch food, and *Aesculus hippocastanum* extract could used as stabilizer in Hemp seed oil Nanoemulsions for biomedical and food applications (Jarzebski et al., 2021). The seeds of *A. chinensis* were used as the source of escin with a clinically significant activity in chronic venous insufficiency, hemorrhoids and post-operative edema (Zhang et al., 2006). However, the leaves and flowers of *A. chinensis* almost be a virgin land for metabolite investigation.

Many plants with high flavonol contents, such as ginkgo leaves, tartary buckwheat, ginseng, soybean, and lotus, are cultivated for the production of healthy foods or herbal teas. Herein, we found 47 flavnoid accumulated in leaves and flowers, especially flavonol glycosides are the main components. Flavonol glycosides are important astringent and bitter substances in teas, due to their extremely low taste thresholds (Sasaki et al., 2014; Guo et al., 2021). Sensory evaluation indicated that the leaves and flowers of *A. chinensis* match the key taste quality of herbal tea.

**Figure 6:**
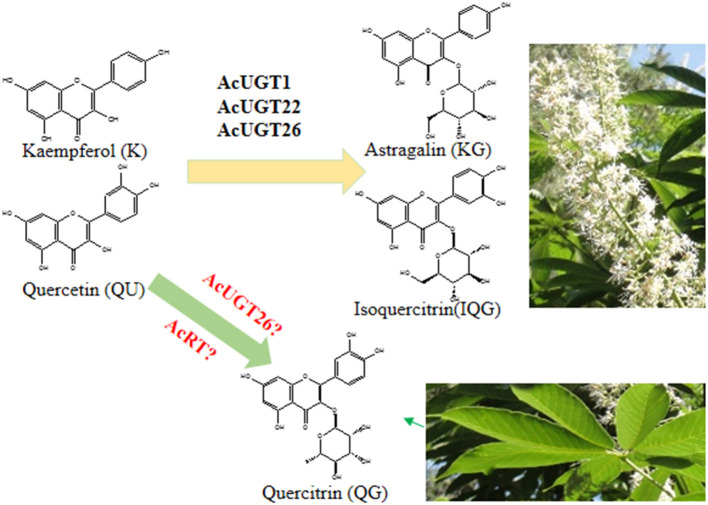
The proposed formation process of astragalin and isoquercitrin in *A. chinensis*.

Flavonol 3-O-glycosides were found to induce a velvety and mouth-coating sensation at very low threshold concentrations, which were far below those of catechins or theaflavins (Scharbert et al., 2004). Consistently, flavonoid profile of *A. chinensis* herbal tea suggested that quercetin 3-O-rhamnose and kaempferol 3-O-glucoside contributed to the taste parameters of the leaves and flowers, respectively. Similar to flavonol 3-O-glycosides in the seeds of *A. turbinate* (Kimura et al., 2017), the flowers and leaves of *A. chinensis* also could be used as food additives, companing with huge inflorescence and lots of leaves as deciduous tree (Figure 6).

### Multi-Dimension Strategy to Reveal the Glucosyltransferases Involved in the Formation of Flavonol Glucosides

Multi-dimension strategy had been developed to reveal the formation of key compounds in food or *planta*, such as oolong tea, *Ziziphora bungeana* and ginseng (Yonekura-Sakakibara et al., 2008; Liu et al., 2018; He et al., 2020; Wu et al., 2020; Yin et al., 2021). The glycosyltransferase that catalyzes flavonol mono-glucosides was inferred by analysis of metabolites and crude proteins activities. The transcriptome data and *in vitro* enzyme activity verification of the recombinant protein showed that AcUGT1 may play an important role in the accumulation of these glycosides in flowers. However, the expression levels of AcUGT22 and AcUGT26 in seeds were about 5-fold greater than that in flowers. Hence, flavonoids may be involved in the catalysis of monoglycosides to polyglycosides in seeds, or their products may be further modified and not reflected in flavonoid profiles, as flavonols reportedly undergo acylation in seeds (Sasaki et al., 2014).

Glycosyltransferase, which catalyzes the formation of glycosides, dictates substrate diversity and specificity (Li et al., 2021). Amino acid alignments and phylogenetic tree analysis showed that UGTs tend to be more regioselective, combined with the enzymatic function *in vitro*. AcUGT22, which was classified to the 3-OGT cluster, catalyzes the formation of flavonol mono-glucosides. Interestingly, AcUGT1 and several other UGTs formed a new family. In addition to 3-glycosylation activities, AcUGT1 may have other catalytic activities, such as a triterpenoid substrate, and thus is a potential candidate to explore the mechanism of escin formation. Similarly, the characteristics of AcUGT26 enzyme activity *in vitro* were not consistent with the evolutionary tree. Although close to the 7-OGT cluster, AcUGT26 and other two AcUGTs formed a unique branch, suggesting that this enzyme may have different catalytic activities.

The elucidation of several plant UGT crystals is the basis for exploring the reaction mechanism of these proteins and secondary metabolites in plants (Li et al., 2007; Yang et al., 2018; Maharjan et al., 2020). The simulation results of the three AcUGTs were all >35% and two were 50%, indicating very high feasibility. Molecular docking showed that sugar donor (UDP-glucose) and sugar acceptor (quercetin) were docked to the pocket of AcUGT1 and AcUGT26, and their location were very close, while the energy of the three mimetic proteins to dock quercetin was the lowest. In the simulation, the hydrogen bonds formed between UDP-glucose and quercetin were mainly located in the N terminal, the PSPG box was close to the C terminal, and several amino acids were in the middle loop. Unlike other reports, the PSPG box was responsible for the interactions between the C terminal and UDPG (Yang et al., 2018). Similarly, Pro12 and Ser15 of AcUGT1 and Ser18 and His21 of AcUGT26 can form hydrogen bonds with UDP-glucose. The docking prediction also found that residues of the PSPG box participated in the interactions of quercetin. Especially, the residues Trp360, Asn361, Glu381, and Gln382 of the PSPG box of AcUGT26 can form hydrogen bonds with the substrate. Due to the lack of UDP-rhamnose standards, molecular docking was used to identify putative UGTs. The docking results of UDP-rhamnose, quercetin, and three AcUGTs showed that the rhamnosyl donor and acceptor were docked to the pocket of AcUGT26, suggesting that the protein might catalyze the rhamnosylation of quercetin (Figure 6).

## Conclusion

Sensory evaluation indicated that flavonol glycosides, which are abundant in the flowers and leaves of *A. chinensis* contribute to the taste of herbal tea. The crude proteins of the flowers and recombinant proteins were screened by differential transcriptome and phylogenetic methods. Enzymatic testing demonstrated that three glycosyltransferases that catalyze the formation of flavonol glucosides were mainly present in the flowers. These findings unraveling the glucosylation of astringency compounds of *A. chinensis* via integrative sensory evaluation, metabolite profiling and enzymatic analysis, could push the utilization of the whole plant in the development of healthy functional products or additives.

## Data Availability Statement

AcUGT1, AcUGT22, and AcUGT26 were named on behalf of the UGT nomenclature committee as UGT71A46, UGT85A121, and UGT90A25 respectively. The datasets presented in this study can be found in online repositories. The names of the repository/repositories and accession number(s) can be found in the article/Supplementary Material.

## Author Contributions

QY, XH, and WS conceived and designed the study. QY, YW, and JC performed the experiments and analyzed the data. QY, JC, and XH wrote the paper. HG analyzed the UGTs information from transcriptome. All authors read and approved the manuscript.

## Funding

This research was supported by Scientific and technological innovation project of China Academy of Chinese Medical Sciences (CACMS Innovation Fund, CI2021A04117, CI2021A05108), Beijing Natural Science Foundation of China (7192138), the National Key R&D Program of China (2019YFC1711100), and the Fundamental Research Funds for the Central Public Welfare Research Institute of China (ZZ13-YQ-097).

## Conflict of Interest

The authors declare that the research was conducted in the absence of any commercial or financial relationships that could be construed as a potential conflict of interest.

## Publisher's Note

All claims expressed in this article are solely those of the authors and do not necessarily represent those of their affiliated organizations, or those of the publisher, the editors and the reviewers. Any product that may be evaluated in this article, or claim that may be made by its manufacturer, is not guaranteed or endorsed by the publisher.

## References

[B1] Cao Q. Q. Zou C. Zhang Y. H. Du Q. Z. Yin J. F. Shi J.. (2019). Improving the taste of autumn green tea with tannase. Food Chem. 277, 432–437. 10.1016/j.foodchem.2018.10.146 30502167

[B2] Chen A. Y. Chen Y. C. (2013). A review of the dietary flavonoid, kaempferol on human health and cancer chemoprevention. Food Chem. 138, 2099–2107. 10.1016/j.foodchem.2012.11.139 23497863 PMC3601579

[B3] Cheng J. T. Chen S. T. Guo C. Jiao M. J. Cui W. J. Wang S. H.. (2018). Triterpenoid saponins from the seeds of *Aesculus chinensis* and their cytotoxicities. Nat. Prod. Bioprospect. 8, 47–56. 10.1007/s13659-017-0148-4 29285602 PMC5803144

[B4] Cui L. Yao S. Dai X. Yin Q. Liu Y. Jiang X.. (2016). Identification of UDP-glycosyltransferases involved in the biosynthesis of astringent taste compounds in tea (*Camellia sinensis*). J. Exp. Bot. 67, 2285–2297. 10.1093/jxb/erw053 26941235 PMC4809296

[B5] Dong F. Hu J. Shi Y. Liu M. Zhang Q. Ruan J. (2019). Effects of nitrogen supply on flavonol glycoside biosynthesis and accumulation in tea leaves (*Camellia sinensis*). Plant Physiol. Biochem. 138, 48–57. 10.1016/j.plaphy.2019.02.017 30849677

[B6] Feng C. Y. Li S. S. Taguchi G. Wu Q. Yin D. D. Gu Z. Y.. (2021). Enzymatic basis for stepwise C-glycosylation in the formation of flavonoid di-C-glycosides in sacred lotus (*Nelumbo nucifera* Gaertn.). Plant J. 106, 351–365. 10.1111/tpj.15168 33486798

[B7] Griesser M. Vitzthum F. Fink B. Bellido M. L. Raasch C. Munoz-Blanco J.. (2008). Multi-substrate flavonol O-glucosyltransferases from strawberry (*Fragaria x ananassa*) achene and receptacle. J. Exp. Bot. 59, 2611–2625. 10.1093/jxb/ern117 18487633 PMC2486459

[B8] Guo X. Y. Lv Y. Q. Ye Y. Liu Z. Y. Zheng X. Q. Lu J. L.. (2021). Polyphenol oxidase dominates the conversions of flavonol glycosides in tea leaves. Food Chem. 339:128088. 10.1016/j.foodchem.2020.128088 32979714

[B9] He J. Yang W. Cheng B. Ma L. Tursunjiang D. Ding Z.. (2020). Integrated metabolomic and transcriptomic profiling reveals the tissue-specific flavonoid compositions and their biosynthesis pathways in *Ziziphora bungeana*. Chinese Med. 15:73. 10.1186/s13020-020-00354-6 32695217 PMC7364582

[B10] Jarzebski M. Smułek W. Siejak P. Rezler R. Pawlicz J. Trzeciak T.. (2021). Aesculus hippocastanum L. as a stabilizer in hemp seed oil Nnanoemulsions for potential biomedical and food applications. Int. J. Mol. Sci. 22:887. 10.3390/ijms22020887 33477381 PMC7830832

[B11] Kapusta I. Janda B. Szajwaj B. Stochmal A. Piacente S. Pizza C.. (2007). Flavonoids in horse chestnut (*Aesculus hippocastanum*) seeds and powdered waste water byproducts. J. Agric. Food Chem. 55, 8485–8490. 10.1021/jf071709t 17867637

[B12] Kimura H. Ogawa S. Ishihara T. Maruoka M. Tokuyama-Nakai S. Jisaka M.. (2017). Antioxidant activities and structural characterization of flavonol O-glycosides from seeds of Japanese horse chestnut (*Aesculus turbinata* BLUME). Food Chem. 228, 348–355. 10.1016/j.foodchem.2017.01.084 28317733

[B13] Li J. Qu G. Shang N. Chen P. Men Y. Liu W.. (2021). Near-perfect control of the regioselective glucosylation enabled by rational design of glycosyltransferases. Green Synth. Catal. 2, 45–53. 10.1016/j.gresc.2021.01.005

[B14] Li L. Modolo L. V. Escamilla-Trevino L. L. Achnine L. Dixon R. A. Wang X. (2007). Crystal structure of *Medicago truncatula* UGT85H2–insights into the structural basis of a multifunctional (iso)flavonoid glycosyltransferase. J. Mol. Biol. 370, 951–963. 10.1016/j.jmb.2007.05.036 17553523

[B15] Liu P. P. Yin J. F. Chen G. S. Wang F. Xu Y. Q. (2018). Flavor characteristics and chemical compositions of oolong tea processed using different semi-fermentation times. J. Food Sci. Technol. 55, 1185–1195. 10.1007/s13197-018-3034-0 29487461 PMC5821678

[B16] Maharjan R. Fukuda Y. Nakayama T. Nakayama T. Hamada H. Ozaki S. I.. (2020). Crown-ether-mediated crystal structures of the glycosyltransferase PaGT3 from *Phytolacca americana*. Acta Crystallogr. D Struct. Biol. 76(Pt 6), 521–530. 10.1107/S2059798320005306 32496214

[B17] Oszmiański J. Kalisz S. Aneta W. (2014). The content of phenolic compounds in leaf tissues of white (*Aesculus hippocastanum* L.) and red horse chestnut (*Aesculus carea* H.) colonized by the horse chestnut leaf miner (*Cameraria ohridella* Deschka and Dimić). Molecules 19, 14625–14636. 10.3390/molecules190914625 25225723 PMC6270754

[B18] Sasaki N. Nishizaki Y. Ozeki Y. Miyahara T. (2014). The role of acyl-glucose in anthocyanin modifications. Molecules 19, 18747–18766. 10.3390/molecules191118747 25405291 PMC6271837

[B19] Scharbert S. Hofmann T. (2005). Molecular definition of black tea taste by means of quantitative studies, taste reconstitution, and omission experiments. J. Agric. Food Chem. 53, 5377–5384. 10.1021/jf050294d 15969522

[B20] Scharbert S. Holzmann N. Hofmann T. (2004). Identification of the astringent taste compounds in black tea infusions by combining instrumental analysis and human bioresponse. J. Agric. Food Chem. 52, 3498–3508. 10.1021/jf049802u 15161222

[B21] Su X. Shen G. Di S. Dixon R. A. Pang Y. (2017). Characterization of UGT716A1 as a Multi-substrate UDP:Flavonoid Glucosyltransferase Gene in *Ginkgo biloba*. Front. Plant Sci. 8:2085. 10.3389/fpls.2017.02085 29270187 PMC5725826

[B22] Sun W. Leng L. Yin Q. Xu M. Huang M. Xu Z.. (2019). The genome of the medicinal plant *Andrographis paniculata* provides insight into the biosynthesis of the bioactive diterpenoid neoandrographolide. Plant J. 97, 841–857. 10.1111/tpj.14162 30444296 PMC7252214

[B23] Wei F. Ma S. C. Ma L. Y. But P. P. Lin R. C. Khan I. A. (2004). Antiviral flavonoids from the seeds of *Aesculus chinensis*. J. Nat. Prod. 67, 650–653. 10.1021/np030470h 15104496

[B24] Wu L. Huang X. Liu S. Liu J. Guo Y. Sun Y.. (2020). Understanding the formation mechanism of oolong tea characteristic non-volatile chemical constitutes during manufacturing processes by using integrated widely-targeted metabolome and DIA proteome analysis. Food Chem. 310:125941. 10.1016/j.foodchem.2019.125941 31835227

[B25] Xu M. Pirtskhalava T. Farr J. N. Weigand B. M. Palmer A. K. Weivoda M. M.. (2018a). Senolytics improve physical function and increase lifespan in old age. Nat. Med. 24, 1246–1256. 10.1038/s41591-018-0092-9 29988130 PMC6082705

[B26] Xu Y. Q. Zhang Y. N. Chen J. X. Wang F. Du Q. Z. Yin J. F. (2018b). Quantitative analyses of the bitterness and astringency of catechins from green tea. Food Chem. 258, 16–24. 10.1016/j.foodchem.2018.03.042 29655718

[B27] Yang M. Fehl C. Lees K. V. Lim E. K. Offen W. A. Davies G. J.. (2018). Functional and informatics analysis enables glycosyltransferase activity prediction. Nat. Chem. Biol. 14, 1109–1117. 10.1038/s41589-018-0154-9 30420693

[B28] Yin Q. Han X. Chen J. Han Z. Shen L. Sun W.. (2021). Identification of specific glycosyltransferases involved in flavonol glucoside biosynthesis in ginseng using integrative metabolite profiles, DIA proteomics, and phylogenetic analysis. J. Agri. Food Chem. 69, 1714–1726. 10.1021/acs.jafc.0c06989 33512142

[B29] Yin Q. Han X. Han Z. Chen Q. Shi Y. Gao H.. (2020). Genome-wide analyses reveals a glucosyltransferase involved in rutin and emodin glucoside biosynthesis in tartary buckwheat. Food Chem. 318:126478. 10.1016/j.foodchem.2020.126478 32126466

[B30] Yin Q. Shen G. Chang Z. Tang Y. Gao H. Pang Y. (2017a). Involvement of three putative glucosyltransferases from the UGT72 family in flavonol glucoside/rhamnoside biosynthesis in *Lotus japonicus* seeds. J. Exp. Bot. 68, 597–612. 10.1093/jxb/erw420 28204516 PMC5444469

[B31] Yin Q. Shen G. Di S. Fan C. Chang Z. Pang Y. (2017b). Genome-wide identification and functional characterization of UDP-glucosyltransferase genes involved in flavonoid biosynthesis in *Glycine max*. Plant Cell Physiol. 58, 1558–1572. 10.1093/pcp/pcx081 28633497

[B32] Yonekura-Sakakibara K. Tohge T. Matsuda F. Nakabayashi R. Takayama H. Niida R.. (2008). Comprehensive flavonol profiling and transcriptome coexpression analysis leading to decoding gene-metabolite correlations in Arabidopsis. Plant Cell 20, 2160–2176. 10.1105/tpc.108.058040 18757557 PMC2553606

[B33] Zhang N. Liu D. Wei S. Cao S. Feng X. Wang K.. (2020a). Phenylethanol glycosides from the seeds of *Aesculus chinensis* var. chekiangensis. BMC Chem. 14:31. 10.1186/s13065-020-00685-3 32337510 PMC7178748

[B34] Zhang N. Wei S. Cao S. Zhang Q. Kang N. Ding L.. (2020b). Bioactive triterpenoid saponins from the seeds of *Aesculus chinensis* Bge. var. chekiangensis. Front. Chem. 7:908. 10.3389/fchem.2019.00908 32039145 PMC6989559

[B35] Zhang Z. Li S. Zhang S. Gorenstein D. (2006). Triterpenoid saponins from the fruits of *Aesculus pavia*. Phytochemistry 67, 784–794. 10.1016/j.phytochem.2006.01.017 16497343

[B36] Zhu C. Peng W. Li Y. Han X. Yu B. (2006). Synthesis of 3-O-(beta-D-xylopyranosyl-(1–>2)-beta-D-glucopyranosyl)-3'-O-(beta-D-glucopyranosyl)tamarixetin, the putative structure of aescuflavoside A from the seeds of *Aesculus chinensis*. Carbohydr Res. 341, 1047–1051. 10.1016/j.carres.2006.02.036 16580652

[B37] Zhuang J. Dai X. Zhu M. Zhang S. Dai Q. Jiang X.. (2020). Evaluation of astringent taste of green tea through mass spectrometry-based targeted metabolic profiling of polyphenols. Food Chem. 305:125507. 10.1016/j.foodchem.2019.125507 31622805

[B38] Zlatanov M. D. Antova G. A. Angelova-Romova M. J. Teneva O. T. (2013). Lipid composition of *Castanea sativa* Mill. and Aesculus hippocastanum fruit oils. J. Sci. Food Agri. 93, 661–666. 10.1002/jsfa.5917 23174908

