# Supplementary material for: Unraveling the Glucosylation of Astringency Compounds of Horse Chestnut via Integrative Sensory Evaluation, Flavonoid Metabolism, Differential Transcriptome, and Phylogenetic Analysis
Source: Front Plant Sci. 2022 Feb 3;12:830343. doi: 10.3389/fpls.2021.830343 (PMC8850972; doi:10.3389/fpls.2021.830343)
Supplement: Supplementary file 3 [file Image_1.PDF]

Supplementary Material

1 Supplementary Data

Supplementary Data 1. The sequences of AcUGTs, primers for clone and transcriptome differential analysis. Note, "/" means uncloned it in next experiments

2 Supplementary Figures

Supplementary Figure 1. The heatmap of flavonoid profile herbal tea madding from *A. chinensis*.

Supplementary Figure 2. The heatmap for AcGTs annotated transcripts in seeds and leaves of *A. chinensis*. F, flowers; R, seeds.

Supplementary Figure 3. The characterization of AcUGTs. A, The amino acids sequence alignment of AcUGTs and others reported UGTs. B, the SDS-Page gel of AcUGTs. C represents UPLC-MS chromatograms of AcUGT recombinant proteins with kaempferol (Ka). D represents UPLC-MS spectrums of AcUGT recombinant proteins with quercetin (Qu).

Supplementary Figure 4. The MS spectrums of products from enzymatic reaction of AcUGTs and flavonol. A&B represent the MS spectrums of enzymatic products from recombinant AcUGT1 protein, UDPG and kaempferol (Ka) or quercetin (Qu). C&D represent the MS spectrums of enzymatic products from AcUGT22, UDPG and Ka or Qu. E&F represent the MS spectrums of enzymatic products from AcUGT26, UDPG and Ka or Qu.

Supplementary Figure 5. The docking of subtrates with AcUGTs. A&B represent the overall and enlarged active domain of docking results from quercetin, UDPG (UDP-glucose) and AcUGT26, respectively. C&D represent the overall and enlarged active domain of docking results from quercetin, UDPR (UDP-Rhamnose) and AcUGT26, respectively. The compound with green sticks indicates UDPG or UDPR, while the compound with yellow sticks represent quercetin.

Figure S1

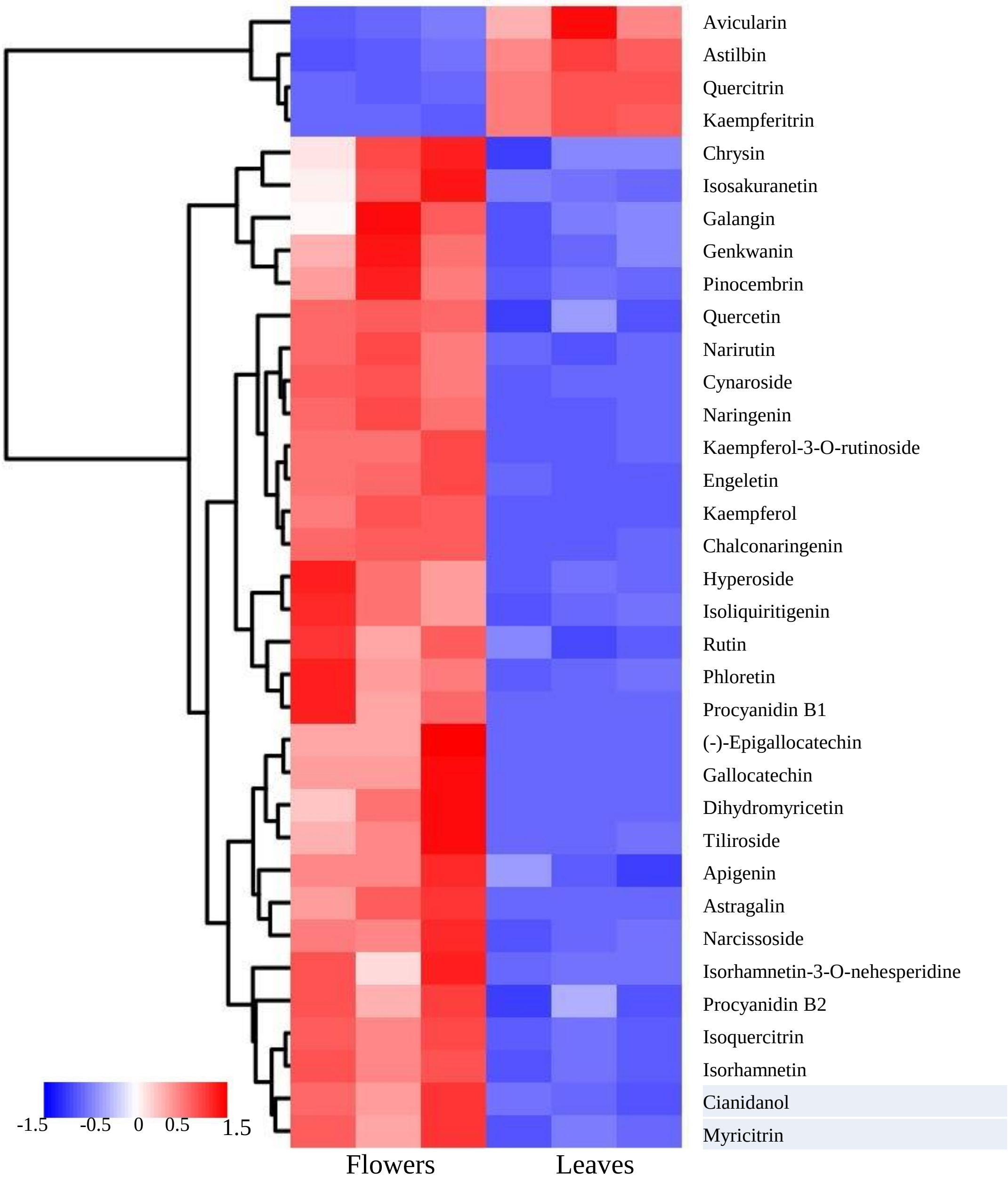

Supplementary Figure 1. The heatmap of flavonoid profile herbal tea madding from *A. chinensis*

Figure S2

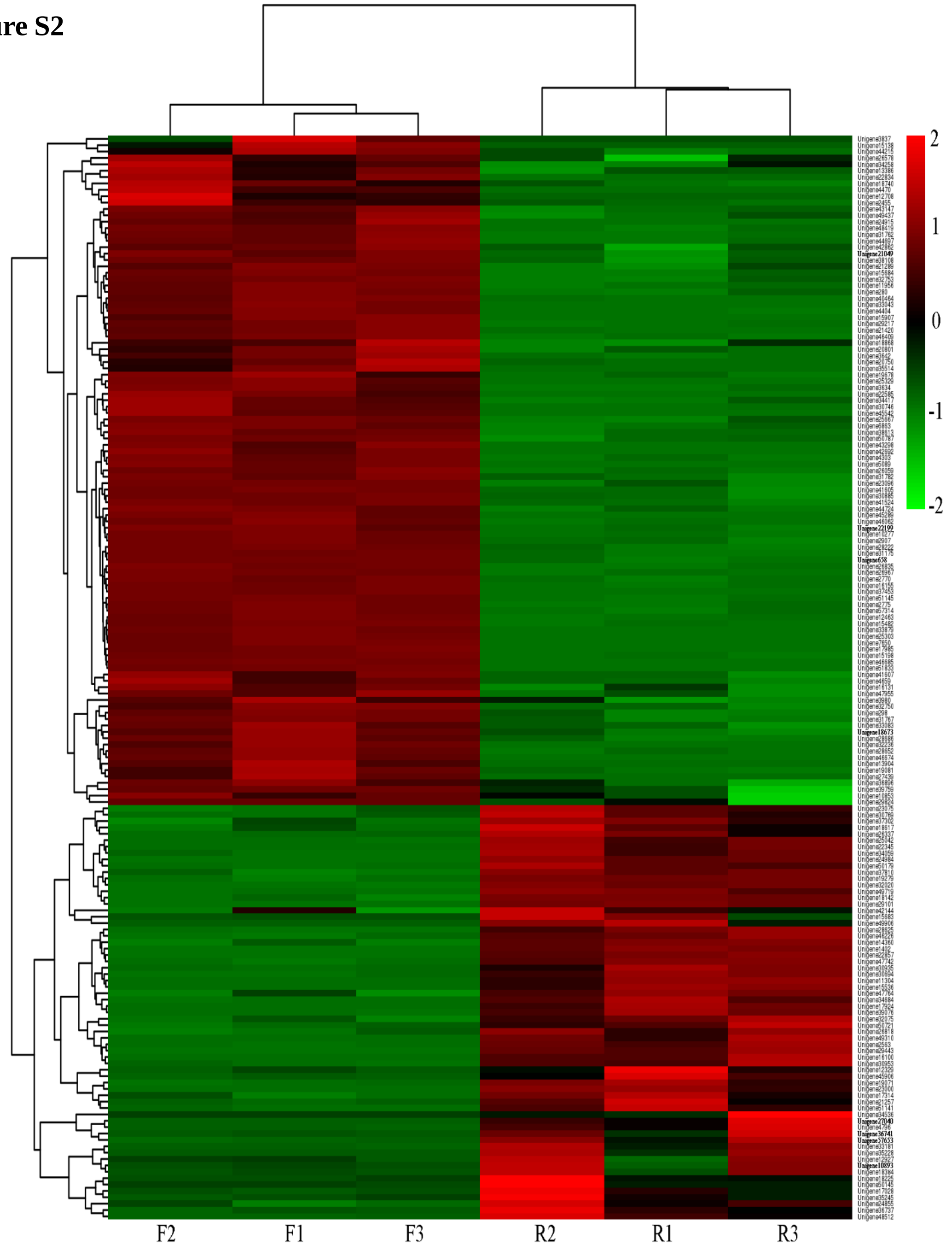

Supplementary Figure 2. The heatmap for AcGTs annotated transcripts in seeds and leaves of *A. chinensis*. F, flowers; R, seeds.

Figure S3

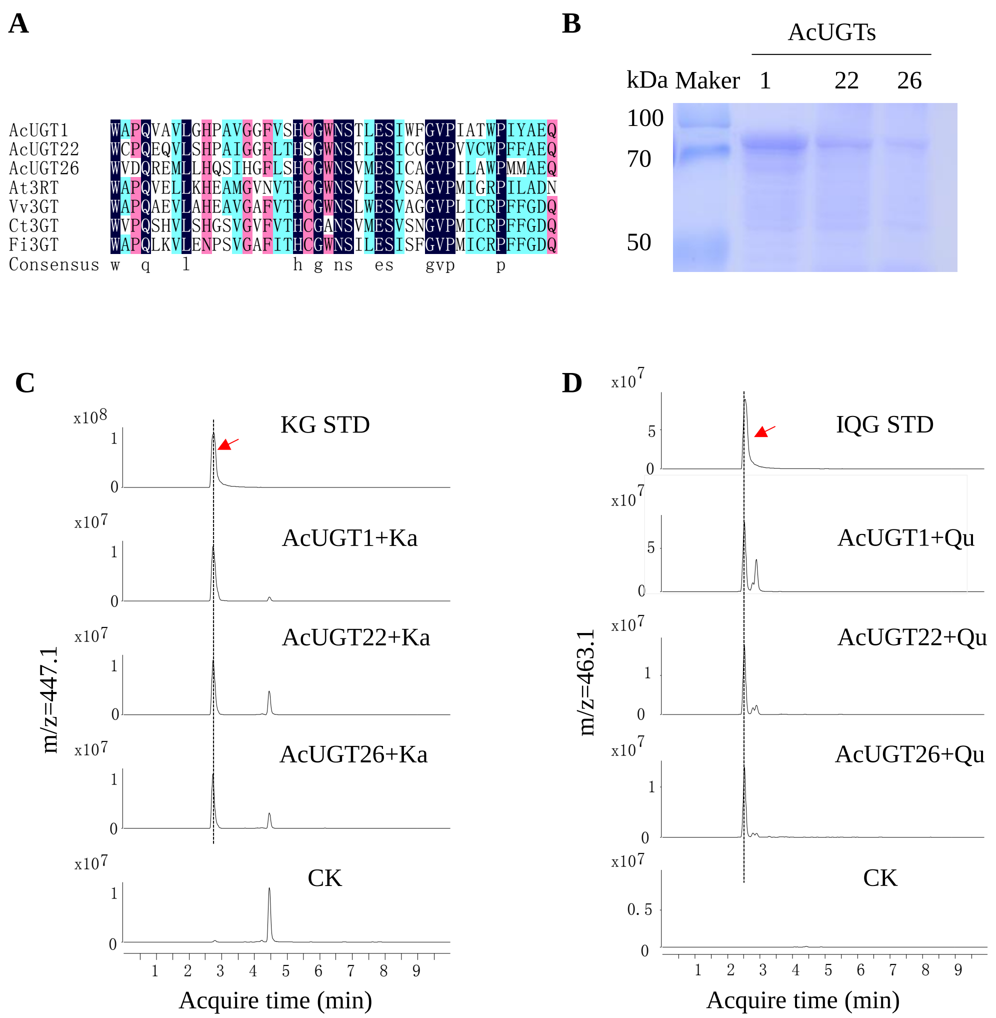

Supplementary Figure 3. T The characterization of AcUGTs. A, The amino acids sequence alignment of AcUGTs and others reported UGTs. B, the SDS-Page gel of AcUGTs. C represents UPLC-MS chromatograms of AcUGT recombinant proteins with kaempferol (Ka). D represents UPLC-MS spectrums of AcUGT recombinant proteins with quercetin (Qu).

Figure S4

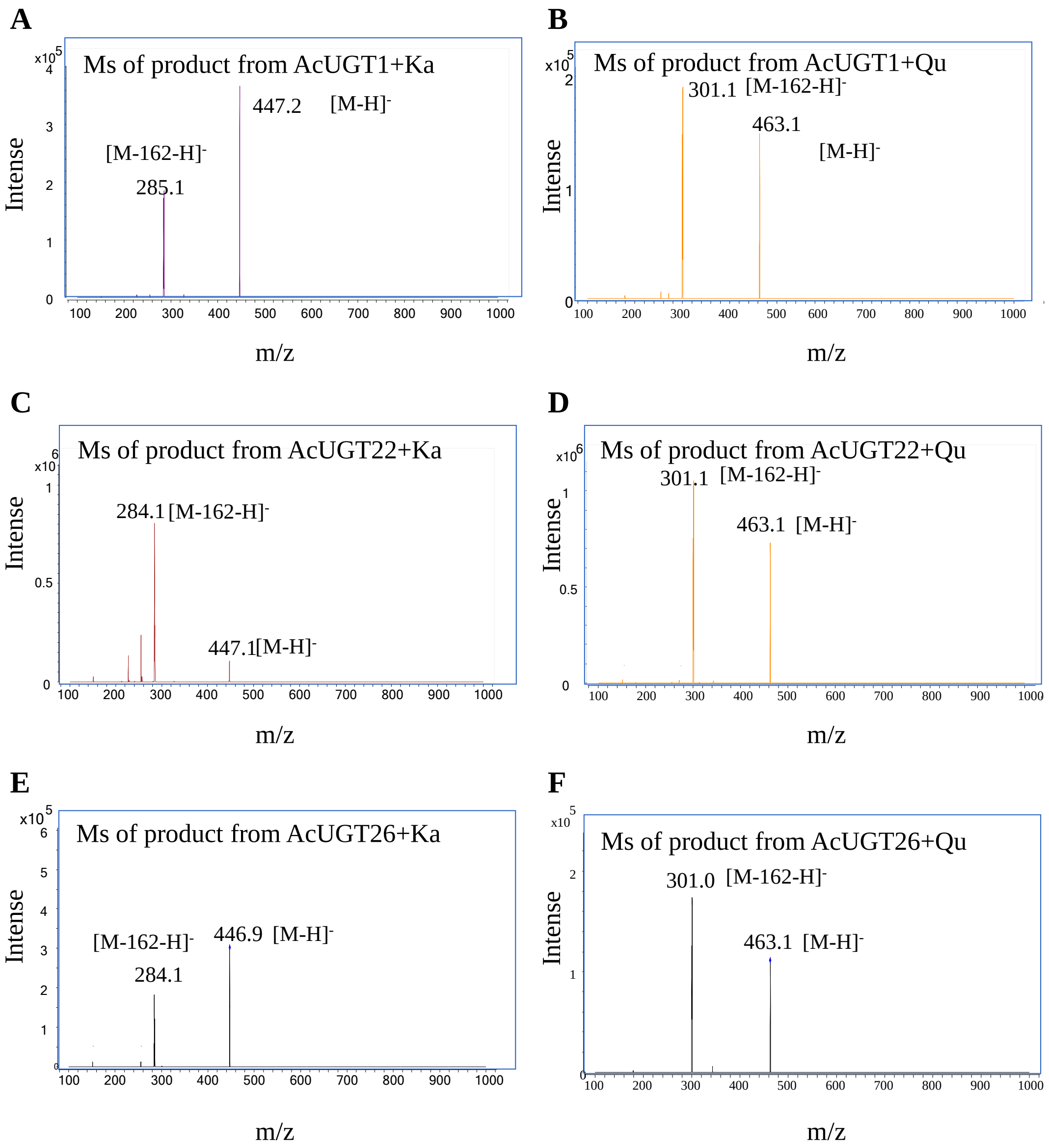

Supplementary Figure 4. The MS spectrums of products from enzymatic reaction of AcUGTs and flavonol. A&B represent the MS spectrums of enzymatic products from recombinant AcUGT1 protein, UDPG and kaempferol (Ka) or quercetin (Qu). C&D represent the MS spectrums of enzymatic products from AcUGT22, UDPG and Ka or Qu. E&F represent the MS spectrums of enzymatic products from AcUGT26, UDPG and Ka or Qu.

Figure S5

A **B**

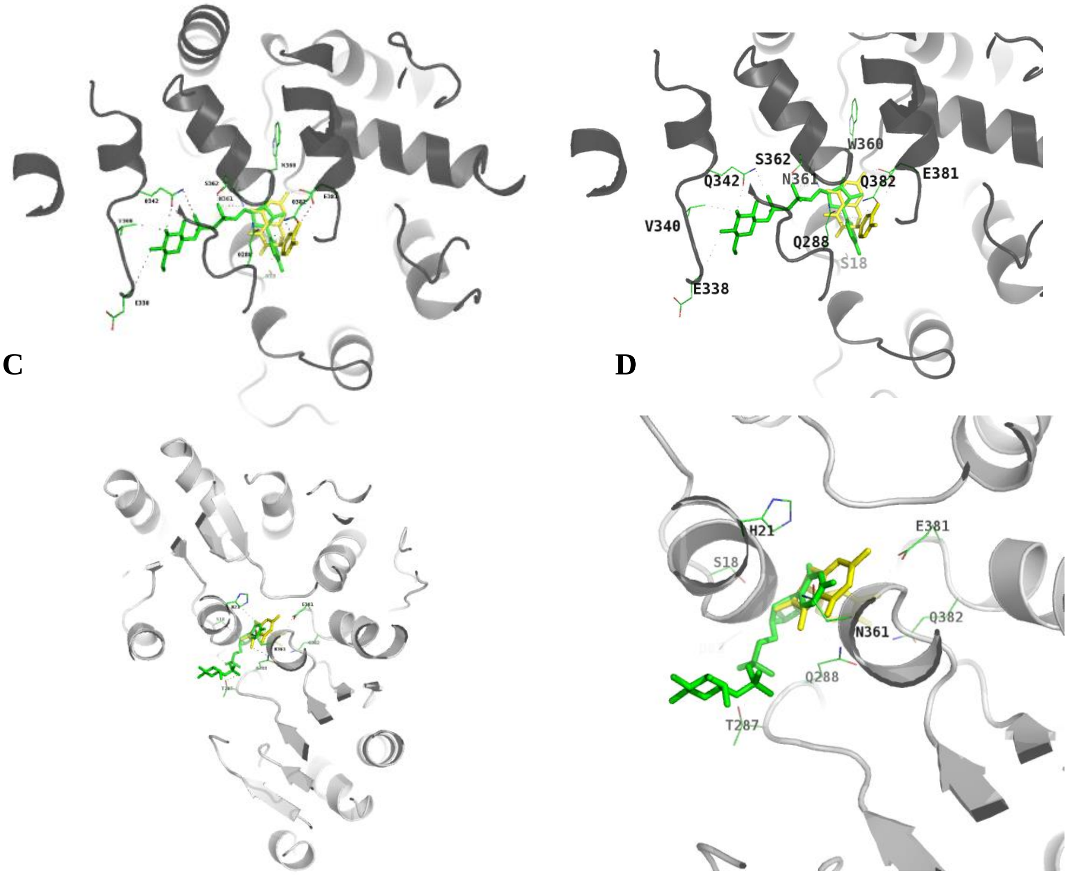

Supplementary Figure 5. The docking of subtrates with AcUGTs. A&B represent the overall and enlarged active domain of docking results from quercetin, UDPG (UDP-glucose) and AcUGT26, respectively. C&D represent the overall and enlarged active domain of docking results from quercetin, UDPR (UDP-Rhamnose) and AcUGT26, respectively. The compound with green sticks indicates UDPG or UDPR, while the compound with yellow sticks represent quercetin.
